# Astaxanthin as a Putative Geroprotector: Molecular Basis and Focus on Brain Aging

**DOI:** 10.3390/md18070351

**Published:** 2020-07-05

**Authors:** Vincenzo Sorrenti, Sergio Davinelli, Giovanni Scapagnini, Bradley J. Willcox, Richard C. Allsopp, Donald C. Willcox

**Affiliations:** 1 Department of Pharmaceutical Pharmacological Sciences, University of Padova, 35131 Padova, Italy; 2 Bendessere™ Study Center, 35131 Padova, Italy; 3 Department of Medicine and Health Sciences “V. Tiberio”, University of Molise, Via de Sanctis s.n.c, 86100 Campobasso, Italy; sergio.davinelli@unimol.it (S.D.); giovanni.scapagnini@unimol.it (G.S.); 4 Department of Geriatric Medicine, John A. Burns School of Medicine, University of Hawaii, Honolulu, HI 96817, USA; willcox@hawaii.edu (B.J.W.); d.willcox@okiu.ac.jp (D.C.W.); 5 Department of Research, Kuakini Medical Center, Honolulu, HI 96817, USA; 6 Department of Anatomy and Reproductive Biology, Institute for Biogenesis Research, John A. Burns School of Medicine, University of Hawaii, Honolulu, HI 96813, USA; allsopp1@yahoo.com; 7 Department of Human Welfare, Okinawa International University, Ginowan 901-2701, Japan

**Keywords:** astaxanthin, geroprotector, longevity, NRF2, FOXO3, SIRT1, neuroprotection

## Abstract

In recent years, the scientific interest in natural compounds with geroprotective activities has grown exponentially. Among the various naturally derived molecules, astaxanthin (ASX) represents a highly promising candidate geroprotector. By virtue of the central polyene chain, ASX acts as a scavenger of free radicals in the internal membrane layer and simultaneously controls oxidation on the membrane surface. Moreover, several studies have highlighted ASX’s ability to modulate numerous biological mechanisms at the cellular level, including the modulation of transcription factors and genes directly linked to longevity-related pathways. One of the main relevant evolutionarily-conserved transcription factors modulated by astaxanthin is the forkhead box O3 gene (FOXO3), which has been recognized as a critical controller of cell fate and function. Moreover, FOXO3 is one of only two genes shown to robustly affect human longevity. Due to its tropism in the brain, ASX has recently been studied as a putative neuroprotective molecule capable of delaying or preventing brain aging in different experimental models of brain damage or neurodegenerative diseases. Astaxanthin has been observed to slow down brain aging by increasing brain-derived neurotrophic factor (BDNF) levels in the brain, attenuating oxidative damage to lipids, protein, and DNA and protecting mitochondrial functions. Emerging data now suggest that ASX can modulate Nrf2, FOXO3, Sirt1, and Klotho proteins that are linked to longevity. Together, these mechanisms provide support for a role of ASX as a potential geroneuroprotector.

## 1. Introduction

Tackling aging is a challenge for any living organism, and longevity in human beings can be defined as a complex "phenotype" [1]. With the increase in human life expectancy, it has become an essential objective to preserve long-term health and maintain organic brain functionality and well-being during aging [1,2]. Healthy aging is the result of the interaction between genes, stochastic processes (such as the unequal partition of cytoplasmatic components and cell-division asymmetry, among others [3]), and the environment, including modifiable lifestyle factors (such as diet and exercise) [3,4,5,6].

Numerous observational studies have shown a positive correlation between the daily use of foods such as fruits and vegetables, nuts, oily fish, and beverages such as tea and red wine, and a large plethora of health benefits [7]. Furthermore, an extensive array of research on several model organisms of aging, (e.g., nematodes, flies, mice), and humans, has shown that some specific phytochemicals, such as resveratrol or spermidine, may counteract the age-related functional decline of cells and tissues and delay the onset of various age-related diseases [8,9,10,11]. These natural compounds may increase cellular resistance to aging by modulating signaling pathways associated with inflammation, autophagy, proteotoxic and oxidative stress, through the upregulation of antioxidant enzymes and sirtuin activity, the inhibition of inflammatory mediators, and by promoting DNA repair and trophic factor production [8,11,12]. 

These compounds have been proposed as “geroprotectors” [13]. A Nobel prize recipient and one of the founders of gerontology, Ilya Mechnikov, coined the term “geroprotector” to indicate a molecule capable of "protecting against aging" both in terms of duration and quality of life [14]. Today, the term has been better characterized by identifying criteria necessary to define a molecule as a geroprotector. The main rule for a molecule to be defined as a geroprotector is the ability to increase lifespan. A geroprotector should also act on molecular, cellular, or physiological biomarkers in order to slow down the progression of age-related changes by improving the quality of life, related to physical, mental, emotional, and social health. Furthermore, the mechanisms of action of a geroprotector should be preserved evolutionarily by increasing an organism’s resistance to unfavorable events [14]. Typically, a geroprotector should exert different molecular mechanisms involved in delaying aging processes. It can act by preventing DNA damage or macromolecule alteration induced by oxidative carbonylation and non-enzymatic glycosylation, acting as metal chelators, ROS-scavengers or anti-amyloid agents [14,15,16,17,18]. A geroprotector can also reduce stress hyper-response, such as the activation of poly (ADP-ribose) polymerase 1 (PARP1), implicated in the acceleration of aging processes linked to hypersecretory, hypertrophic, and pro-inflammatory senescent cells [19,20]. Moreover, molecules reducing hyperactivated pro-aging proteins, such as insulin-like growth factor 1 (IGF1), mammalian target of rapamycin (mTOR), nuclear factor kappa-light-chain-enhancer of activated B cells (NF-κB), cyclooxygenase 2 (COX2), inducible nitric oxide synthase (iNOS) or enhancing anti-aging proteins such as nuclear factor erythroid 2-related factor 2 (Nrf2), sirtuin 1 (Sirt1), Klotho, and the forkhead box (FOX) protein family can be suitable to be considered as geroprotectors [14,20,21,22,23]. For example, rapamycin and resveratrol, which inhibit mTOR, extend lifespan in some animals [24,25]. 

Among the various naturally derived molecules that are candidates to be considered geroprotectors, astaxanthin (ASX) is particularly promising [26]. Thanks to its chemical-physical characteristics and potential brain tropism, ASX has strong potential as a novel geroneuroprotector [27]. Although the exact mechanisms by which ASX promotes these effects are still to be clarified, several studies have highlighted its ability to stimulate various biological mechanisms at the cellular level, including the modulation of transcription factors and genes directly linked to longevity-related processes. One of the principal evolutionarily-conserved transcription factors modulated by astaxanthin is the forkhead box O3 (FOXO3) gene, which has been recognized as an important controller of fate and function of neural cells [28]. This review summarizes the latest evidence on the role of ASX as a potential geroneuroprotector with a particular emphasis on the regulation of Nrf2, FOXO3, Sirt1, and Klotho proteins linked to longevity.

## 2. Carotenoids, Diet, and Longevity

Astaxanthin belongs to the vast phytochemical family of carotenoids, a group of natural tetraterpenoid pigments distributed widely in plants, algae, fungi, and bacteria. Carotenoids are normally classified into carotenes and xanthophylls according to the oxygenation degree. These compounds are insoluble in water and contain a long polyene central chain of conjugated double bonds that functions as a chromophore, with maximal absorption at 400–500 nanometers, responsible for their typical yellow to orange/red coloration. Carotenoids play different and critical roles in phytobiology and are thought to have strongly conditioned the evolution of life on this planet [29]. 

In plants, carotenoids have a critical photoprotective role by quenching triplet state chlorophyll molecules and scavenging singlet oxygen (and other toxic oxygen species) formed within the chloroplast—they also act as signaling molecules to mediate plant development and responses to several other environmental cues and provide precursors for the biosynthesis of phytohormones [30]. Apart from their central functions in plants, some carotenoids play relevant roles in animals, as essential precursors of retinol (vitamin A precursors). Other carotenoids do not support vitamin biosynthesis, but they are still able to promote optimal health during the life course and have been proposed as “putative vitamins”. 

They are also known to be very efficient physical and chemical quenchers of singlet oxygen (^1^O2), as well as potent scavengers of other reactive oxygen species, even playing an important role as nutritional antioxidants. In general, animals are unable to produce their carotenoids, and therefore, the only way to obtain these compounds is from the diet. More than 700 types of carotenoids have been found from natural sources thus far. In the human diet, carotenoid sources are mostly fruit and vegetables. Six carotenoids are present in the American diet accounting for 95% of the carotenoids found in human blood: lutein, zeaxanthin, α and β-carotene, lycopene, and β-cryptoxanthin [31]. Astaxanthin is a marine carotenoid, and it is present in many fish, crustaceous, and edible seaweeds. Thus, in humans, it is common in diets containing abundant marine foods, such as in the Japanese, in the Inuit and some coastal countries of the Mediterranean area. 

Population studies have highlighted the relevant role of carotenoids on human health, and their potential influence on aging and longevity. There is good evidence that carotenoids help optimize a healthy lifespan, and a low dietary intake of these compounds has been associated with several degenerative age-related diseases. Low total plasma carotenoid level has been significantly associated with all-cause mortality and mortality by cancer in men after controlling for the main potential confounding factors [32]. High carotenoid plasma levels are negatively correlated with inflammatory and aging-related diseases and positively correlated with physical performance [33]. Higher total plasma carotenoids were associated with significant protection against the decline in walking speed, and the development of severe walking disability in older adults from the Invecchiare in Chianti (Aging in the Chianti Area (InCHIANTI)) study [34]. In a study of serum carotenoids in 13,000 Americans in the National Health and Nutrition Examination Survey (NHANES) survey, an increase in all-cause mortality was associated with a low level of total carotenoids [35]. A more recent study has shown in US adults that increasing levels of blood carotenoid were significantly associated with longer leukocyte telomeres [36]. The strength of the evidence varies, but there is an increasing interest in the role of carotenoids in the human diet for achieving healthy longevity. The hypothetical protective role of carotenoids could come from their antioxidant properties. Carotenoid supplementation has been found to improve antioxidant status and reduce lipid peroxidation in humans [37]. Beyond the antioxidant potential, most of these compounds appear to have several different molecular targets, impinging on different signaling pathways and showing pleiotropic activity on cells and tissues. A possible general mechanism of carotenoid healing activity, correlated to their ability to overexpress highly protective inducible genes, is their involvement in the modulation of signaling pathways associated with inflammation, autophagy, proteotoxic and oxidative stress [31]. More specifically, examples of signaling pathways affected by carotenoids include the upregulation of antioxidant enzymes, sirtuins, DNA repair efficiency, trophic factor production, and inhibition of inflammatory mediators [38]. Compared to the other carotenoids, ASX, due to its peculiar chemical structure, is endowed with additional abilities to tune molecular pathways specifically involved in longevity enhancement.

## 3. Astaxanthin: From a Diet Component to an Anti-Aging Supplement

Ketocarotenoid astaxanthin, 3,3′-dihydroxy-β,β-carotene-4,4′-dione, is synthesized in nature by several species of microalgae and phytoplankton, and by yeast as *Xanthophyllomyces dendrorhous*. It is produced in the cytoplasm of these organisms and functions mainly to counter oxidative stress. Microalgae produce astaxanthin in cytosolic lipid bodies under environmental stress or adverse culture conditions, such as high light, high salinity, and nutrient deprivation. It is produced in the cytoplasm of these organisms and functions mainly to counter oxidative stress. Microalgae produce ASX in cytosolic lipid bodies under environmental stress or adverse culture conditions, such as high light, high salinity, and nutrient deprivation. Phytoplankton and microalgae are consumed in the marine food chain, and as a result, ASX is accumulated in the body of several species of fish, crustaceans and a few aquatic bird species (e.g., flamingo), eliciting the pinkish-red color in their flesh, shells, and feathers [39]. ASX is typically present in salmon, trout, red snapper, tilapia, crab, shrimp and lobster, octopus, and squid, and many other kinds of seafood and freshwater fish. Wild-caught salmon is a good source of ASX with contents reported in the range of 26–38 mg/kg flesh. ASX content in farmed Atlantic salmon was reported as 6–8 mg/kg flesh. ASX is also available in the European (6 mg/kg flesh) and the Japanese market (25 mg/kg flesh) from large trout. Shrimp, crab, and salmon can serve as dietary sources of astaxanthin. To ingest 4 mg of ASX through one’s diet, one should eat between 180 and 200 grams of salmon per day [39]. 

The traditional Okinawan diet, known for healthy aging properties, is rich in marine-based carotenoids such as ASX and fucoxanthin due to the high intake of seaweeds (macroalgae) that include wakame (*Undaria pinnatifida*), kombu (*Laminaria japonica*), and hijiki (*Hijikia fusiformis*), among others, as well as other seafood such as fish and crustaceans [40]. These foods are very low in caloric density but high in nutrient density, and several have been shown to act as caloric restriction (CR) mimetics [41].

The green microalga *Hematococcus pluvialis* has exceptional pigment accumulation potential under stress conditions, and it is one of the best sources of natural ASX for the food and pharma industries. Microalgae-derived ASX is generally recognized as safe by the FDA, meaning it can be sold as a dietary supplement. In Europe ASX-rich oleoresin from *Haematococcus pluvialis* algae is authorized in food supplements at levels of up to 40–80 mg/day which corresponds to a maximum authorized level of 8 mg ASX per day, considering the acceptable daily intake for the total ASX derived from supplement and the background diet being 0.2 mg/kg BW. In recent years, the scientific interest in this molecule has grown exponentially, moving from the use as a food coloring to a promising anti-aging molecule [39]. The peculiarity of its action is to be found in its chemical structure. By virtue of the central polyene chain containing 13 conjugated double bonds, ASX acts as a scavenger of free radicals in the internal membrane layer and simultaneously controls oxidation on the surface of the membrane itself through the end rings [42]. These characteristics are the main reasons for the exceptional antioxidant capacity of ASX, which is approximately ten times more effective than β-carotene or lutein and about 100 times than α-tocopherol [43]. In a randomized, blinded, four-arm, prospective study in 32 healthy individuals aged 60–70 years with confirmed signs of oxidative stress, a lysosomal formulation of dark chocolate containing 7 mg of co-crystalized ASX with enhanced bioavailability, showed interesting effects on the correction of oxidative status in aging individuals, suggesting a promising synergic combination of ASX with dark chocolate [44].

As a supplement, ASX has been implicated in numerous health benefits in humans, including a reduction in cardiovascular diseases, enhancement of the immune response, and reduction in the occurrence of various cancers [45]. 

A very successful field of ASX nutraceutical application is skin anti-aging and photoprotection, recently reviewed by our group [46]. The effects of ASX on wrinkle formation reduction, hyperpigmentation suppression, and photoaging inhibition have been reported in several clinical studies [46]. 

Aging is also characterized by a progressive loss of muscle mass and a delay in post-exercise recovery [47,48]. ASX has been widely studied in vivo models for its potential role in preventing muscle loss and ameliorate muscle strength and exercise performance, and there is preliminary evidence in endurance performance and post-exercise recovery in humans [49]. In healthy older adults, in particular, a recent study has shown how supplementation with ASX has improved muscle strength and muscle size of cross-sectional area (CSA), in addition to the increase in endurance and distance covered with physical training alone [50]. Therefore, ASX supplementation, in combination with a functional training program, can potentially improve muscle strength, endurance, and mobility in the elderly.

One of the most fascinating fields of astaxanthin geroprotector use is surely in neuroprotection, which is reviewed in detail in Section 4.

Collectively, ASX is a promising geroprotective molecule capable of preserving organic physiological functions for healthy aging. The geroprotective role of ASX is due to its ability to modulate various signal pathways related to longevity. In the following section, the main biochemical pathways involved in the geroprotector role of astaxanthin are analyzed.

## 4. Major Biochemical Pathways Involved in Astaxanthin Pro-Longevity Mechanisms

Astaxanthin is a powerful antioxidant with translational protective effects against various human diseases and physiological disorders. As a potent antioxidant, ASX has been thought to play important roles in alleviating oxidative stress-driven adverse physiological effects during aging and by this increasing lifespan in cells and model organisms. Lifespan extension and anti-aging effects of astaxanthin in *Caenorhabditis elegans, Drosophila melanogaster,* and mice were strongly associated with decreased oxidative stress [51,52]. *Drosophila melanogaster* mutants in CuZn-superoxide dismutase (SOD1) and Mn-superoxide dismutase (SOD2), as well as mutant-lines with reduced levels of SOD1, SOD2 and catalase, show significant lifespan extension and amelioration of age-related decline in motility when fed with ASX rich *Haematococcus pluvialis* [53]. In mice, ASX treatment was shown to prevent age-related decreased saliva secretion [53]. In fact, the antioxidant activity of ASX prevented ROS generation and accumulation, increased the functionality of aquaporins(AQPs)5 by improving the organizational structure of the salivary glands, and limited the functional decline of salivary glands related to the aging process [52].

**Figure 1:**
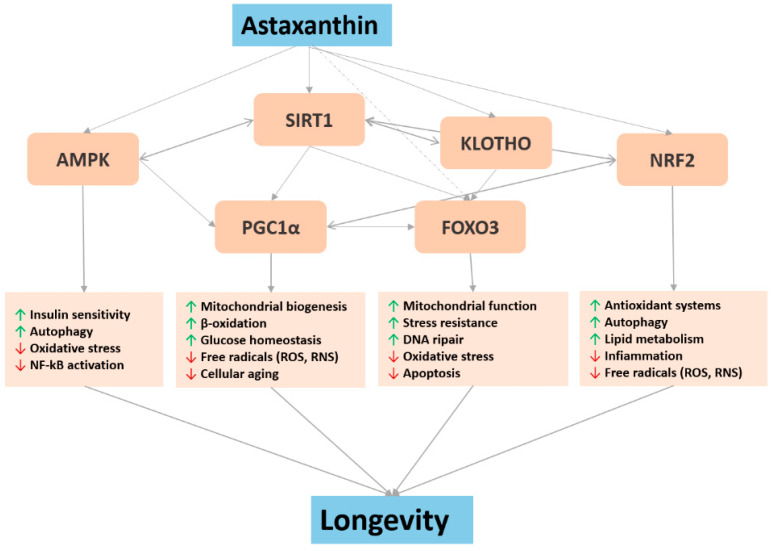
Potential molecular mechanisms involved in astaxanthin geroprotective effects.

The critical action of ASX on redox biochemistry is almost certainly due to its peculiar chemical structure and its exceptional quenching and scavenging abilities. However, the mechanisms underlying its antioxidant functions in cells are not fully understood. In addition to directly interacting with free radicals, ASX has been shown to inhibit oxidative damage by activating several key genes related to longevity and aging (Figure 1). Such genes and their encoded proteins are included in a variety of signaling pathways, i.e., nutrient sensing insulin/IGF1, oxidative stress and antioxidant defense, and the control of immune-inflammatory responses. 

### 4.1. Nrf2 and NF-κB

In naturally long-lived species and experimental longevity models, a profound cytoprotective response constitutively upregulated, characterized by greater resistance to stress, is normally highlighted, and it is primarily regulated at the molecular level by the cytoprotective multifunctional regulator nuclear factor erythroid 2-related factor 2 (Nrf2) [54]. Nrf2, encoded by *Nfe2l2* gene, is a transcription factor responsible for the regulation of cellular redox balance and protective antioxidant and phase II detoxification responses in mammals [21]. The Nrf2-signaling pathway regulates different cytoprotective pathways by activating the transcription of more than 200 genes related to oxidative stress and inflammatory modulation, the metabolism of drugs and toxins, as well the stability of proteins via proteasomal degradation or autophagy [21]. Nrf2 controls the basal and induced expression of an array of antioxidant response element-dependent genes, including glutamate-cysteine ligase (GCL), thioredoxin reductase 1 (Txnrd1), NAD(P)H-quinone oxidoreductase 1 (NQO1) and heme oxygenase-1 (HMOX1), through binding to the antioxidant response element (ARE) binding sequence [55]. Under homeostatic conditions, Nrf2 is bound to the inhibitory protein Keap1 within the cytosol. Alteration to the conformation of Keap1, however, by various oxidants and electrophiles leads to the liberation of Nrf2 and its translocation to the nucleus where it binds to ARE. This activates a cascade of events which, in the end, affects the oxidative status of the cells and provides robust protection against oxidative challenge. Nrf2 is considered not only as a cytoprotective factor regulating the expression of genes coding for anti-oxidant, anti-inflammatory, and detoxifying proteins, but it is also a powerful modulator of species longevity. Nrf2 has been suggested as “a guardian of healthspan and gatekeeper of species longevity” [55]. In a study performed in ten rodent species, ranging in maximum lifespan potential (MLSP) between 4 and 31 years, it was shown that in the long-lived naked mole rats, there is a constitutively high level of Nrf2 in comparison to other rodent species with shorter lifespans [56]. Recent years have seen a strong interest in nutraceuticals that can stimulate endogenous antioxidant enzymes. It has been well established that bioactive food agents such as resveratrol, curcumin, and epicatechins promote cell survival by enhancing the activity of Nrf2 and the expression of its downstream cytoprotective enzymes [57]. ASX exerts various cytoprotective effects by promoting Nrf2/ARE signaling. In a mouse model of cardiac injury, induced by ochratoxin A, ASX played a protective role on the myocardium through the activation of the Nrf2/ARE signaling pathway, together with modulation of the mitochondria-mediated apoptosis pathway [58].

In another study of human umbilical vein endothelial cells (HUVEC), the effects of ASX on the production of reactive oxygen species (ROS) and the activity of antioxidant enzymes, as well as the nuclear factor erythroid 2-related factor 2 (Nrf-2)/heme oxygenase-1 (HO-1) pathways were examined. The results suggested that ASX activates the antioxidant pathway Nrf-2/HO-1 by modulating ROS production [59].

In a rat model of streptozotocin-induced diabetes, the nephroprotective potential of ASX on decreasing excessive oxidative stress and the accumulation of fibronectin in mesomeric glomerular cells challenged with high glucose(HG) was assessed. ASX treatment alleviated metabolic parameters, renal morphology, and extracellular matrix accumulation. Moreover, ASX treatment strongly promoted the nuclear translocation and transcriptional activity of Nrf2, as well as the upregulation of the expression of SOD1, NQO1 and HO-1, ultimately extinguishing the highest level of ROS and inhibiting HG-induced fibronectin, intercellular adhesion molecule 1 (ICAM1) and transforming growth factor beta 1 (TGFβ1) expression. Collectively, these data suggested a nephroprotective effect of ASX on diabetic nephropathy, mainly related to the activation of Nrf2/ARE signaling [60].

In cigarette smoke-induced emphysema in mice, the protective effect of ASX through the activation of the Nrf2 pathway was evaluated. Higher expression levels of Nrf2 and HO-1 were detected in lung homogenates of ASX-fed mice. In addition, the number of inflammatory cells in the bronchoalveolar lavage fluid was significantly reduced and emphysema was significantly suppressed in the ASX groups. In conclusion, ASX protects against oxidative stress by activating the Nrf2 pathway and improves cigarette smoke-induced emphysema [61].

Taken together, ASX exerts a large number of protective effects in different cellular phenotypes by promoting Nrf2 signaling.

A plethora of studies have also demonstrated that there is an interplay between the Nrf2 system and the NF-κB signaling network, which is the principal pathway involved in the modulation of inflammatory responses [62]. The increased activity of NF-κB during aging is associated with enhanced production of pro-inflammatory cytokines. The role of Nrf2 against inflammation has been related to its ability to antagonize NF-κB. A number of carotenoids have been shown to reduce NF-κB activity. In particular, astaxanthin has been shown to inhibit the activity of IκB kinase, preventing the phosphorylation and degradation of IκB inhibitory proteins, and subsequently the translocation of the NF-κB p65 subunit to the nucleus, suppressing cytokine expression in different cellular models [62,63].

In peripheral blood mononuclear cells in pre- and post-partum Murrah buffaloes, ASX supplementation blocked nuclear translocation of NF-κB p65 subunit and IκBα degradation, which correlated with its inhibitory effect on IκB kinase (IKK) activity [64].

Similarly, in a hamster model of oral cancer, ASX inhibited NF-κB and Wnt/β-catenin signaling pathways via inactivation of Erk/MAPK and PI3K/Akt to induce intrinsic apoptosis inhibiting the development of hamster buccal pouch carcinomas [65].

An in vitro work on an astrocyte stretch injury model explored the molecular mechanism of ASX-mediated inhibition of the Na-K-Cl cotransporter 1 (NKCC1), an intrinsic membrane protein activated by various insults that can induce brain edema. ASX pretreatment significantly prevented the trauma-induced initiation of NF-κB, the subsequent pro-inflammatory cytokine expressions and cell apoptosis [66].

Collectively, these date emphasize how ASX anti-inflammatory properties also correlate with the inhibition of the NF-κB pathway.

### 4.2. FOXO

The forkhead box O3 gene (FOXO3), and its homologs in model organisms, belongs to a family of transcription factors (FoxOs) associated with the regulation of genes involved in inflammation, metabolism, oxidative stress, inflammation, and DNA protection, among other roles [67,68]. The FoxO factors are regulated by several signal transduction cascades. The main regulator of FoxO function is the phosphoinositide 3-kinase (PI3K) pathway, whereas FoxO function is ‘fine-tuned’ by the protein casein kinase 1 (CK1) and the dual-specificity regulated kinase 1A (DYRK1A) pathway. These kinases regulate the intracellular localization and function of FoxO proteins by phosphorylating FoxO factors within several different domains. The role of FoxO3 in longevity can be linked directly to the ability of this transcription factor to regulate aspects of cellular function, such as metabolism, resistance to oxidative stress, DNA damage, autophagy, and programmed cell death. 

Recently, a local FOXO3-centric gene network located on chromosome 6 was discovered by Donlon and colleagues that provides additional insight as to the mechanism of this gene’s actions [69]. They performed DNA sequencing of chromosome 6 in long-lived individuals and found that forty-one FOXO3 SNPs were associated with longevity. Thirteen of these SNPs had predicted alterations in transcription factor binding sites and formed a “gene neighborhood”, connected through distant, long-range physical contact points to 46 neighboring genes. The neighborhood appears to be in physical contact, via RNA polymerase II binding chromatin looping, with sites in the FOXO 3 promoter, and may function as a *cis*-regulatory unit. The gene neighborhood acts via CCCTC-binding factor zinc finger protein (CTCF ) binding sites, over a 7.3 Mb distance, on chromosome 6q21. Under cellular stress, FOXO3 physically moves toward neighboring genes and activates the neighborhood with consequent effects. As with FOXO3, the 46 neighboring genes act together to modify cell resilience processes, including stress response, energy/nutrient sensing, cell proliferation, autophagy, apoptosis and stem cell maintenance that optimize healthy aging. 

The FOXO3 response to environmental stimuli is likely a critical factor in mitigating aging and age-related diseases, including cardiovascular diseases, type II diabetes, various cancers, and neurodegenerative diseases. FOXO3, therefore, represents a potential target in a therapeutic intervention aimed at promoting healthy aging and prolonging lifespan. FOXO3 represents a key gene in the insulin/IGF-1 pathway, influencing lifespan across diverse species. In humans, several single nucleotide polymorphisms (SNPs) in the FOXO3 gene have been associated with longevity in different populations [22,67]. The link between the insulin/IGF-1 signaling pathway and longevity was first identified in the nematode *C. elegans*, where the effect of mutations in DAF-2, the homolog of the insulin receptor, on lifespan was shown to be dependent on its downstream effector, DAF-16, the ortholog of human FOXO3 [38]. Importantly, calorie restriction, which is unequivocally considered the best strategy for promoting healthy aging and longevity, also robustly activates DAF-16 and FOXO3, and blocking the activity of FOXO3 abrogates the longevity-enhancing effect of caloric restriction [70]. Furthermore, there are also nutraceutical compounds capable of inducing FOXO3, and these substances could theoretically help improve health and increase lifespan. ASX has been shown to efficiently activate daf-16 and extend *C. elegans* lifespan. Specifically, the addition of ASX in the diet was able to increase longevity in both wild-type N2 and long-lived mutant *age-1* of *C. elegans* by up to 30%—this would translate to about 30 years in humans. In the same study, a mutant strain of *C. elegans* devoid of DAF-16 did not exhibit any improvement in longevity following addition of ASX to the diet, thus demonstrating that the effect of life extension induced by ASX, at least in *C. elegans*, is connected to the activation of DAF-16 (i.e., the FOXO3 ortholog). The addition of ASX to the diet of wild-type and *age-1* animals mainly enhanced the mRNA expression of the DAF-16 target genes and increased the nuclear localization of the DAF-16 transcription factor. Furthermore, it was shown that ASX also caused a decrease in mitochondrial ROS production during the long-term exposure to these animals [38,53]. A recent study has shown that ASX upregulates FOXO3 expression in the renal tubular epithelium, and is able to efficiently protect it in an in vitro and in vivo model of iohexol-induced acute kidney injury [71]. Recently, our research team at the John A. Burns School of Medicine of the University of Hawaii has shown that astaxanthin "activates" the FOXO3 gene in mice, with the detection of an increase of about 90% of its expression in the heart tissue. In this pilot study, mice were fed either normal food or food containing a low or high dose of ASX for 2 weeks, followed by the analysis of FOXO3 expression levels in the brain, skeletal muscle, blood, and heart tissues. The animals that were fed the higher amount of the ASX compound experienced a significant increase in the activation of the FOXO3 gene in their heart tissue, with a similar, though more modest, activation in the blood as well [72].

### 4.3. Sirtuin1 and Klotho

Silent mating type information regulation 2 homolog (SIRT)1 and KL (Klotho) are two longevity genes involved in delaying cellular senescence and extending lifespan through the regulation of multiple anti-aging cellular processes [23,73,74,75]. In humans, sirtuin 1, also known as NAD-dependent deacetylase sirtuin 1, is a member of the sirtuin family of proteins and it is encoded by the SIRT1 gene [76]. Sirtuin1 is an enzyme that deacetylates proteins that contribute to reaction to stressors and anti-aging mechanisms. Suppression of cellular senescence by sirtuin 1 is mainly mediated through delaying age-related telomere attrition, sustaining genome integrity and promotion of DNA damage repair [77]. In addition, sirtuin 1 modulates the organismal lifespan by interacting with several lifespan regulating signaling pathways, including the insulin/IGF-1 signaling pathway, AMP-activated protein kinase, and FOXO [76,77,78]. 

Klotho is an enzyme that in humans is encoded by the KL gene. Klotho is involved in many biological pathways in both humans and animals and appears to have major effects throughout our lifespan. Klotho influences longevity, memory, and cognition [79,80,81], kidney function, and slows the progression of diabetes and cancer [75,82,83,84]. 

*Klotho*^–/^^–^ mice exhibit a premature aging phenotype with a significantly shortened life span characterized by multiple human-resembling disorders including atherosclerosis, vascular and kidney disease, osteoporosis, cognitive impairment, and infertility [82,85]. Further, *Klotho* gene expression was found to be decreased in the liver and kidney during aging [86,87].

The search for sirtuin1 and klotho activators is one of the most extensive and robust topics of anti-aging research. Some hopes are put on natural compounds, including astaxanthin. Preliminary studies are unveiling potential astaxanthin geroprotector activity through the modulation of *Sirt1* and *Klotho* gene expression.

In a mouse model of transverse aortic constriction (TAC)-induced myocardial fibrosis, the protective potential of astaxanthin on cardiac function and post-operative fibrosis was assessed. In addition, the acetylation and phosphorylation of small mother against decapentaplegic receptor-activated (R-SMAD) and the expression and activity of SIRT1 were evaluated to determine a potential protective mechanism of astaxanthin. The results showed how ASX improved heart function and attenuated fibrosis. The increases induced by TAC surgery in the expression of phosphorylated and acetylated R-SMAD were attenuated by ASX treatment as well as the translocation and transcriptional activity of R-SMAD. In addition, ASX induced the expression and activity of SIRT1. These results demonstrate that ASX improved cardiac function and attenuated fibrosis and SIRT1 induction contributed to the ASX protective mechanisms [88].

Another in vivo study evaluated the protective effect of ASX on acute contrast-induced renal injury (CI-AKI) in 40 adult male rats, analyzing a possible protective mechanism of astaxanthin mediated by the SIRT1-p53 pathway. In the ASX-treated group, a significant increase in SIRT1 expression levels and a decrease in p53 expression levels (P 0.05) were demonstrated. ASX has overall demonstrated a protective effect on CI-AKI, and the mechanism appears to be potentially related to the SIRT1-p53 pathway [89].

The effect of ASX on sirtuin1 and klotho was also assessed in an accelerated aging model consisted of mice chronically treated with a combination of d-galactose and jet lag. Six weeks of ASX treatment protected liver weight loss and improved muscle endurance of the elderly mice. These results have been associated with a reduction in serum oxidative stress and an increase in the activity of antioxidant enzymes. In addition, ASX reversed the reduced expression pattern of sirtuin1 and klotho. In this study, ASX supplementation prevented liver weight loss, improved locomotive muscle function, and exerted significant anti-aging properties by reducing oxidative stress and improving the expression of age-related genes [90].

Taken together, ASX anti-aging properties may be partly related to the regulation of sirtuin1 and klotho activity. 

## 5. Astaxanthin: A Putative Geroneuroprotector?

Astaxanthin has recently been studied as a putative neuroprotective molecule able to preserve brain aging in different experimental models of brain damage or neurodegenerative diseases. Dietary ASX is taken up by many tissues in rats. Research has found that ASX unique chemical structure allows it to readily cross the blood–brain barrier [91] and reach the brain in significant concentrations in rats [92]. Thus, the brain is considered an important target organ of ASX [93]. 

From a kinetic point of view, numerous studies have validated ASX’s ability to cross the blood–brain barrier and to concentrate significantly on the central nervous system. ASX has been observed to alleviate brain aging in rats by attenuating oxidative damage to lipids, protein, and DNA and by restoring the activities of antioxidant enzymes, including glutathione peroxidase and superoxide dismutase [94,95,96]. 

In 2010, an in vitro study investigated the effects of ASX on stem cell potentiation through an increased proliferation of neural progenitor cells (NPCs). ASX treatment significantly increased the proliferation and formation of NPC colonies. In particular, ASX induced significant activation of phosphatidylinositol 3-kinase (PI3K) and its downstream mediators in a time-dependent manner. ASX also upregulated some proliferation-related transcription factors (Rex1, cyclin-dependent kinase 1 (CDK1) and 2), coupled with overexpression of stemness genes (octamer-binding transcription factor 4 (OCT4), SRY (sex-determining region Y)-box 2 (SOX2), Nanog, and kruppel-like factor 4 (KLF4)) for the acquisition of active self-renewal activity [97]. 

A recent study evaluated the effect of ASX on cognitive function and neural plasticity in young and elderly mice. One month supplementation with ASX improved cognitive performance in the hippocampus and increased long-term potentiation in older mice [96].

Another in vivo study analyzed the effect on spatial memory on mice fed a diet containing 0.02, 0.1, or 0.5% of ASX [98]. The highest ASX dosages (0.1 and 0.5%) showed a positive correlation to neurogenesis processes and survival of new neurons in the hippocampus, one of the most relevant brain areas in memory consolidation. Although the mechanisms underlying this phenomenon have yet to be fully deciphered, it still seems that ASX is an effective activator of BDNF, a fundamental neurotrophic factor in the engraftment and survival of new neurons [98,99]. ASX’s action on neuronal plasticity could explain the improved cognitive functions and learning tests found in an in vivo experiment on animal models in which ASX was administered [99]. ASX thus alleviates brain aging by restoring brain-derived neurotrophic factor (BDNF) levels in both the brains and hippocampus in rats [99].

By studying the subcellular localization of ASX at a neuronal level in an in vitro model, it was observed how this molecule accumulates specifically at the level of cell membranes and mitochondria [100]. Mitochondrial efficiency is crucial for the survival of neurons, and in neurodegenerative diseases, there is a gradual impairment of mitochondrial efficiency with an increase in oxidative stress [101]. Mitochondrial decoupling and free radical production cause substantial damage to mitochondria, leading to mitochondrial insufficiency [102]. ASX, since it can actually cross the blood–brain barrier [92], is a potential preservation compound of neural function also through the protection of mitochondrial functionality [99,102]. In a recent study, pretreatment with ASX in mouse neural progenitor cells stimulated with H2O2 inhibits apoptotic cell death by promoting cell growth instead [103]. The mechanisms of action mediated by ASX are related to the recovery of mitochondrial ATP production and to the blocking of cytochrome c release through the activation of the signaling of the mitogen-activated protein kinase (MEK) and the induction of the anti-apoptotic protein Bcl-2 [103]. Another study evaluated the neuroprotective effect of ASX on oxidative stress-induced toxicity in primary cultures of cortical neurons and on brain damage induced by focal cerebral ischemia-reperfusion in rats [104]. ASX pretreatment significantly inhibited H2O2-induced apoptosis and restored the mitochondrial membrane potential (MMP) on primary cortical neuronal cultures. In vivo, on the other hand, ASX prevented ischemic brain injury induced by middle cerebral artery occlusion by improving neurological deficit in a dose-dependent manner [104]. Moreover, in cell models associated with Parkinson’s, where neuronal toxicity was induced by 6-hydroxydopamine (6-OHDA), or by 1-methyl-4-phenyl 1,2,3,6-tetrahydro pyridine (MPTP), ASX prevented neuronal death by inhibiting mitochondrial apoptotic cascade [105,106]. ASX also effectively reduced neurotoxicity in cellular models of Alzheimer’s disease. On neurons cultured with β-amyloid fibrils, ASX neuroprotective action was related to the activation of the transcription factor Nrf2 and to the induction of the antioxidant enzyme heme oxygenase-1(HO-1) [107].

A recent in vivo study in a rat model of d-galactose-induced aging, evaluated the protective effect of ASX (15 mg/kg) on oxidative brain damage and possible mechanisms of action through the analysis of serum metabolic profiles [108]. The results showed that ASX significantly increased the catalase (CAT), SOD and glutathione peroxidase (GSH-Px) activities by 26%, 30%, and 53%, respectively. ASX treatment also significantly increased the mitochondrial membrane potential by 18% compared to the control group and the activities of the respiratory chain complexes I and IV by 50.17% and 122.87%, respectively. In addition, reduced age-related morphological changes in the cerebral cortex and hippocampus by ASX were also found. In serum metabolic profiles, ASX induced an increase in the levels of N-acetyl-L-leucine, N-acetyl-L-tyrosine, and methionine sulfoxide, which are relevant for protecting nerve cells.

Moreover, ASX decreased the levels of iossoxicolic acid and chenodeoxycholic acid through the biosynthetic pathway of primary biliary acid, with a reduction in cerebral mitochondrial dysfunction. In conclusion, ASX seems to ameliorate brain antioxidant defenses, mitochondrial activity, and mitochondrial respiratory chain and improve neuronal damage, finally exerting a neuroprotective role [108].

**Figure 2:**
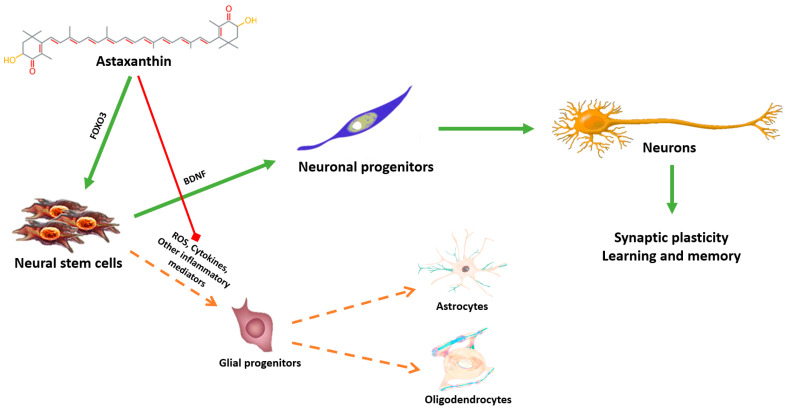
Astaxanthin potentially guides neural stem cells towards differentiation to neurons both through the stimulation of the forkhead box O3 gene (FOXO3) and the brain-derived neurotrophic factor (BDNF) and through the inhibition of oxidative and chronic inflammatory processes that instead favor the formation of new glia.

Another potential and promising target mediating ASX neuroprotective mechanism is represented by the evolutionarily-conserved FOXO family of transcription factors which has emerged as significant judge of neural cell fate and function [28]. FOXO3 modulates neuronal cell survival, cell signaling and stress responses in the progression from neural stem cells to mature neurons in both physiological and pathological conditions [28]. It is also involved in other processes related to neurogenesis, brain antioxidant systems, neurotransmitter synthesis, and acts as a guardian of neuronal integrity by inhibiting age-progressive axonal degeneration in mammals [109]. A recent paper demonstrated a direct effect of FOXO3 in the genomic network for maintaining a healthy mammalian stem cell pool to support lifelong neurogenesis [110]. ASX can thus potentially guide stem neural cells towards differentiation to neurons both through the stimulation of FOXO3 and BDNF and through the inhibition of oxidative and chronic inflammatory processes that instead favor the formation of new glia (Figure 2).

In humans, to date, a few studies have started to explore ASX beneficial properties on cognitive processes. In a pilot study, male subjects suffering from mild cognitive impairment (MCI) were divided in three groups and supplemented with ASX-rich *Haematococcus pluvialis* extracts equivalent to 4 mg (Group A), 8 mg (Group B), or 20 mg (Group C) of Ax dialcohol contained in soft capsules, once daily for 4 weeks. Group C supplemented with 20 mg/day ASX for 12 weeks showed a significant improvement in cognitive performance measured through the CogHealth and P300 tests [111]. Similar results were obtained in a randomized, double-blind, controlled trial in men and women between 45 and 64 years of age, with mild cognitive decline, who were given a ASX-rich *Haematococcus pluvialis* extract containing 6 mg or 12 mg/day of ASX for 12 weeks. Already at the lower dosage, ASX improved cognitive performance measured through the CogHealth and Groton Maze Learning Test [112]. In another study, the effects of a composite supplement containing food-derived antioxidants, specifically astaxanthin and sesamin(AS), were evaluated on cognitive function in people with MCI. The results showed a significant improvement in psychomotor speed and processing speed in the AS group compared to placebo measured with the central nervous system vital signs (CNSVS) test, and suggested an improvement in cognitive function with daily AS supplementation [113].

## 6. Conclusions

Although human data are limited to clinical trials and other research on age-related disease and disability, not longevity, this research review supports the possibility that astaxanthin is a potential geroprotector capable of affecting the rate of human aging and promoting longevity. Over the past ten years, significant progress has been made in understanding that low-grade chronic inflammation and redox imbalance are critical factors for the development of aging-related diseases. Despite the "translational" gap between basic research and clinical research—often related to misuse of phytochemicals as a class of equal antioxidants without considering their different characteristic in terms of bioavailability, mechanism of action, and tropism in the body—the current understanding of the molecular interactions between phytochemical compounds and inflammatory and oxidative responses could help in the design of effective and personalized nutritional strategies to delay the onset of chronic diseases and promote healthy aging. In this context, our laboratory and others have highlighted the importance of specific food-derived phytochemicals such as astaxanthin for maintaining the efficiency of nutrient-sensing longevity pathways, a crucial mechanism for achieving healthy longevity. The geroneuroprotective properties of astaxanthin appear, at least in part, attributable to the reduction in oxidative stress, inflammation, and the neuronal mitochondrial function, as well as to the improvement of the dysregulation of gene expression related to aging and to the promotion of neurogenesis processes. In conclusion, more research is needed, but astaxanthin appears to be a potentially promising new geroneuroprotector.
